# Sample-Specific Generalized Cross-Validation for Gene Network Analysis of Cytarabine Response in Cancer Cell Lines

**DOI:** 10.3390/ijms27188261

**Published:** 2026-09-16

**Authors:** Jooee Oh, Heewon Park

**Affiliations:** 1 Department of Statistics, Sungshin Women’s University, Seoul 02844, Republic of Korea; 2 School of Mathematics Statistics and Data Science, Sungshin Women’s University, Seoul 01133, Republic of Korea; 3 Data Science Center, Sungshin Women’s University, Seoul 01133, Republic of Korea; 4 M&D Data Science Center, Institute of Science Tokyo, Bunkyo-ku, Tokyo 113-8510, Japan; 5 Human Genome Center, Institute of Medical Science, University of Tokyo, 4-6-1 Shirokane-dai, Minato-ku, Tokyo 108-8639, Japan

**Keywords:** gene network, sample specific analysis, model evaluation, generalized cross validation

## Abstract

Sample-specific gene regulatory network analysis can reveal molecular heterogeneity associated with individual characteristics, such as anticancer drug sensitivity. The varying coefficient model with kernel-based $L_{1}$ regularization enables the estimation of such networks, but its performance depends strongly on hyperparameter selection. Conventional cross-validation is computationally intensive and provides only an averaged evaluation across samples, limiting its suitability for sample-specific analysis. To address these limitations, we propose doubleS-GCV, a sample-specific generalized cross-validation criterion for selecting hyperparameters in sample-specific gene network estimation. DoubleS-GCV provides a separate model evaluation for each sample while substantially reducing computational burden. Monte Carlo simulations demonstrated that doubleS-GCV achieved accurate gene selection and network estimation and outperformed conventional information criteria, including AIC, BIC, AICC, and HQC. Application to GDSC cancer cell lines identified Cytarabine sensitivity-specific gene networks and candidate biomarkers supported by previous studies. The estimated networks also exhibited nonlinear structural changes across Cytarabine sensitivity levels, indicating that molecular interactions vary with drug response. These results demonstrate that doubleS-GCV provides an efficient and reliable model selection framework for sample-specific gene network analysis.

## 1. Introduction

Gene network analysis is crucial for understanding complex mechanism involved in diseases that are often driven by intricate interactions among multiple genes. In order to effectively estimate gene network, various computational strategies have been developed and applied to uncover complex mechanism of diseases [1,2,3]. However, most network inference techniques construct a single population-level gene network that fails to capture the sample-specific regulatory structures of individual samples. However, each sample has its own unique gene network, because molecular interplays vary across individual samples. Therefore, a single aggregate network cannot accurately describe the sample-specific molecular interplays and may fail to capture biological heterogeneity at the individual level.

To address this issue, Shimamura et al. [4] proposed a computational strategy, called NetworkProfiler, based on the varying coefficient model with the kernel-based $L_{1}$-type regularization. The kernel-based $L_{1}$-type regularization method assigns higher kernel weights to samples with modulator values similar to those of the target cell line, whereas dissimilar samples receive lower kernel weights. This allows NetworkProfiler to capture biological heterogeneity across samples more accurately. The sample-specific analysis based on the kernel-based $L_{1}$-type regularization heavily relies on the hyperparameters, i.e., two shrinkage parameters for the $L_{1}$-type regularization and a bandwidth parameter of the kernel function. Thus, the hyperparameters selection is a crucial issue in sample-specific gene network analysis. Cross-validation (CV) has often been used as a model selection criterion [5]. However, the CV has critical limitations. First, it imposes a high computational burden owing to repeated model evaluations and estimations. Second, the ordinary CV provides an averaged model evaluation result across all samples, and thus the CV is not suitable for sample-specific gene network analysis. Several model selection approaches can be used to determine the hyperparameters of regularized network models [6]. AIC balances model fit and complexity with an emphasis on predictive performance, whereas BIC imposes a stronger complexity penalty and generally favors sparser models. AICc introduces a finite-sample correction to AIC, making it more suitable when the sample size is limited relative to model complexity, while HQC employs a different complexity penalty from those of AIC and BIC. For high-dimensional model selection, EBIC adds a model-space penalty to BIC [7], and EHQC extends the HQC penalty to more strongly control model complexity. Stability Selection provides an alternative resampling-based approach that selects variables according to their reproducibility across subsamples [8]. However, these criteria and procedures do not provide model evaluation tailored to each target sample. Moreover, resampling-based approaches, including conventional cross-validation and Stability Selection, require repeated model estimation and may become computationally expensive in sample-specific network analysis. These limitations motivate the development of an efficient sample-specific model evaluation criterion.

**Figure 1:**
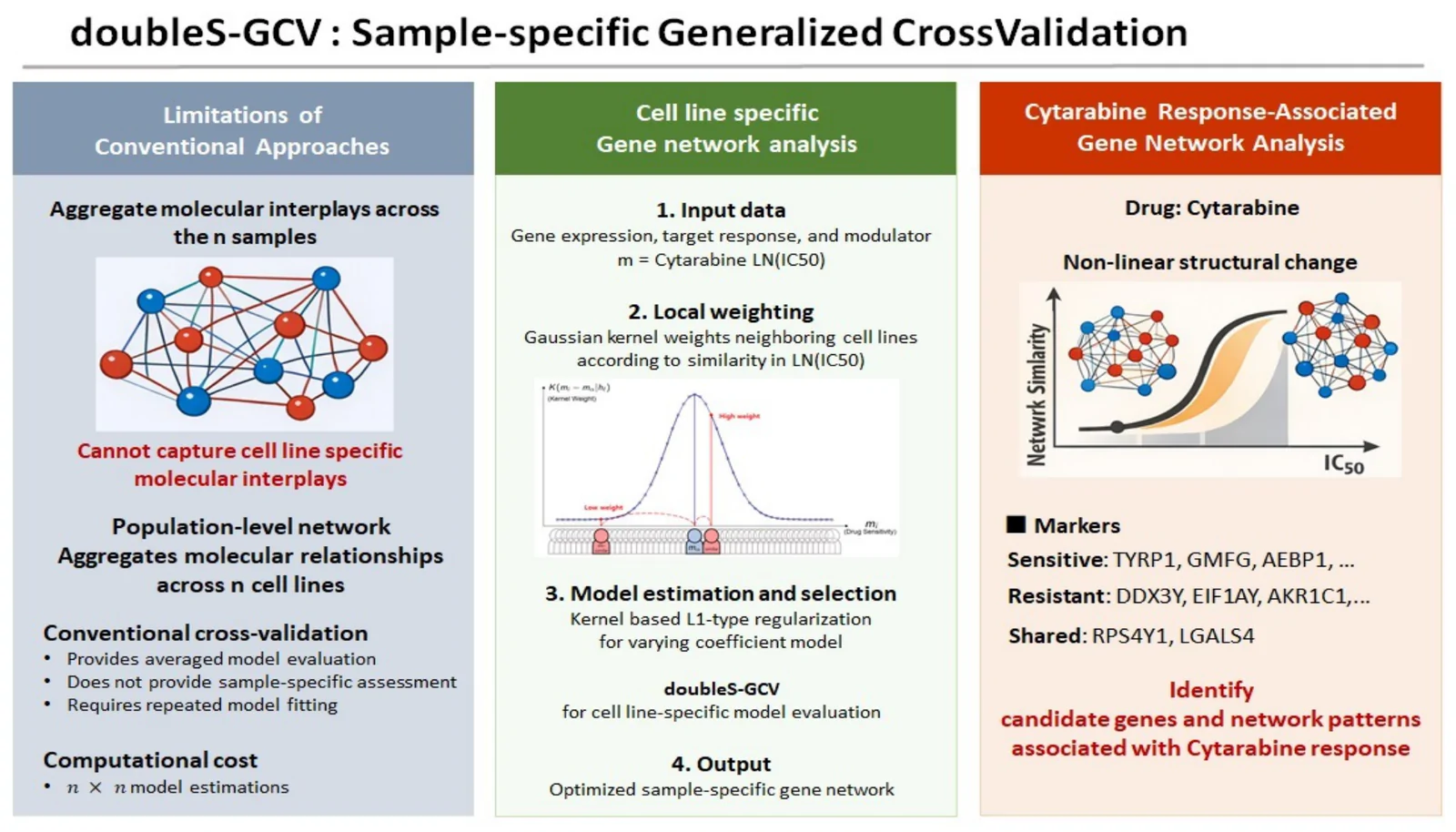
Overview of the doubleS-GCV framework and its application to Cytarabine response-associated gene network analysis. The left panel summarizes the limitations of conventional population-level network analysis and cross-validation, which aggregate molecular relationships and model-evaluation results across samples and require repeated model fitting. The middle panel illustrates the four main steps of doubleS-GCV: (1) input of gene expression, target response, and modulator values; (2) Gaussian-kernel weighting of cell lines according to similarity in Cytarabine LN(IC50); (3) estimation of a kernel-weighted elastic-net varying coefficient model and sample-specific evaluation of 27 hyperparameter combinations using doubleS-GCV; and (4) construction of an optimized sample-specific gene network. The right panel illustrates the comparison of network structures across Cytarabine sensitivity levels and the identification of sensitive-specific, resistant-specific, and shared candidate genes and network patterns.

In this study, we propose a novel model evaluation criterion, called sample-specific Generalized Cross-Validation (doubleS-GCV), for sample-specific gene network analysis. Figure 1 shows a graphical overview of the proposed framework. We first represent the objective function of sample-specific gene network estimation (i.e., kernel-based $L_{1}$ type regularized regression model) without the kernel function. We then derive the generalized cross-validation (GCV) from the cross-validation criterion in line with [6]. In the derivative of GCV, the objective function of the kernel-based $L_{1}$-type regularized regression model should be differentiable to compute the hat matrix. However, the objective function cannot be differentiable, because of the $L_{1}$-norm penalty. To settle on this issue, we refer to the local quadratic approximation (LQA) of the $L_{1}$-type penalty [9] and then derive GCV for sample-specific gene network analysis. Through simulation studies, we demonstrated that the proposed doubleS-GCV achieves superior performance for gene network analysis, and computational efficiency compared to existing model evaluation criteria, making it especially suitable for large-scale, personalized gene network analysis. We then applied the proposed method to Genomics of Drug Sensitivity in Cancer (GDSC) dataset. Notably, our strategy successfully identified Cytarabine sensitivity-specific gene networks and identified candidate biomarkers associated with Cytarabine-sensitive and Cytarabine-resistant cell lines, respectively. Furthermore, we demonstrated that gene network structures change nonlinearly with the anti-cancer drug sensitivity, suggesting that the molecular interplays vary depending on the drug sensitivity of cell lines.

The remainder of this paper is organized as follows: In Section 4, we introduce the computational strategy for sample-specific gene network estimation and detail the development of sample-specific GCV. We present the evaluation results from the simulation studies in Section 2.1. We then describe the results of the Cytarabine sensitivity-specific gene network analysis in Section 2.2. Conclusions are provided in Section 3.

## 2. Results

We conducted simulation studies to evaluate the performance of the proposed doubleS-GCV in the viewpoint of (1) computational efficiency and (2) accuracy in gene selection and network estimation for sample-specific gene network analysis. Because doubleS-GCV is a model evaluation criterion, all comparisons were conducted using the same underlying network estimation model, thereby isolating the effect of the model selection criterion. We compared the performance of our method with other model selection criteria, including AIC, BIC, AICC, and HQC.

### 2.1. Simulation Study

#### 2.1.1. Evaluation Result 1: Computational Efficiency

To formally derive the time complexity of our proposed method, let *n* be the number of samples (n=100 in this evaluation), *q* be the number of potential regulator genes (q=200,500,and1000), *G* be the total size of the hyperparameter grid encompassing $\gamma_{l\alpha },\delta_{l\alpha }$, and $h_{l\alpha }$, and C(n,q) be the computational cost for a single estimation of the kernel-based $L_{1}$-type regularized regression. The traditional leave-one-out cross-validation (LOO-CV) requires a nested loop structure where, for each target cell line α (*n* times) and each hyperparameter combination *G*, the model must be re-estimated by leaving out each observation in turn (*n* times), leading to a total complexity of $T_{LOOCV}=O\left(n\times G\times n\times C\left(n,q\right)\right)=O\left(n^{2}\cdot G\cdot C\left(n,q\right)\right)$. Similarly, the 10-fold CV requires 10 re-estimations per grid point, resulting in a complexity of $T_{\mathrm{10-fold}\;\mathrm{CV}}=O\left(n\times G\times 10\times C\left(n,q\right)\right)$. In contrast, our proposed doubleS-GCV utilizes the hat matrix $H_{l\alpha }$ derived through local quadratic approximation (LQA), allowing the model to be estimated only once per grid point and target sample. Consequently, the total complexity is reduced to $T_{\mathrm{doubleS-GCV}}=O\left(n\times G\times 1\times C\left(n,q\right)\right)=O\left(n\cdot G\cdot C\left(n,q\right)\right)$, which is theoretically equivalent to the efficiency of information-theoretic criteria such as AIC and BIC while achieving a significant computational speedup compared to both LOO-CV and 10-fold CV. Table 1 summarizes the computation time (in seconds) required to estimate all models using different model selection criteria. Notably, doubleS-GCV, AIC, and BIC provide computationally efficient results compared with CV-based approaches.

The results indicate that the ordinary CV is not suitable for sample-specific analysis that requires *n* time estimations for each sample. In contrast, doubleS-GCV serves as an effective model selection criterion that significantly reduces computational burden. It implies that doubleS-GCV is well suited for large-scale or high-dimensional gene network analysis.

#### 2.1.2. Evaluation Result 2: Performance Under Linear Coefficient-Function Settings

We evaluated the accuracy of doubleS-GCV in terms of edge selection and edge weight estimation. Table 2 summarizes the averaged performance metrics (T.P, T.N, the average of T.P and T.N, MSE, and AUROC) across 100 simulation datasets.

In addition to the conventional information-theoretic criteria (AIC, BIC, AICc, and HQC), we further included EBIC [7], an extended Hannan–Quinn criterion (EHQC), and Stability Selection [8] as additional competitors. The proposed doubleS-GCV demonstrates consistently strong performance across all four types of varying coefficient functions. In terms of variable selection, doubleS-GCV generally provides a favorable balance between T.P and T.N, achieving the highest average of these two measures for Types 1 and 3 and competitive values for Types 2 and 4. Its AUROC values also remain consistently high across all scenarios, indicating stable discrimination between true and irrelevant regulatory edges. Most notably, doubleS-GCV yields the smallest MSE for every coefficient-function type, demonstrating its clear advantage in accurately estimating edge weights and the underlying gene network structure. Overall, doubleS-GCV exhibits superior performance in network estimation and strong overall variable-selection performance across the considered scenarios.

#### 2.1.3. Evaluation 3: Performance Under Challenging Simulation Settings

Table 3 presents the results under the challenging simulation setting characterized by a smaller sample size, a sparse coefficient structure, increased noise, and block-correlated predictors. Across all four coefficient-function types, doubleS-GCV achieved the highest T.P rates, the lowest MSE values, and the highest AUROC values. These results indicate that doubleS-GCV effectively identified true regulatory edges while maintaining accurate edge-weight estimation under the more challenging data structure. Stability Selection produced the highest T.N rates and Avg values, reflecting its stronger tendency to exclude irrelevant variables; however, this was accompanied by lower T.P rates and substantially larger MSE values. AIC showed performance close to that of doubleS-GCV for several metrics, whereas BIC, EBIC, and EHQC generally yielded higher T.N but lower T.P and AUROC values. Overall, doubleS-GCV maintained a favorable balance between edge detection and estimation accuracy across all four coefficient-function types, supporting its stable performance under sparse, noisy, and correlated predictor settings.

Taken together, comparison with the original setting indicates that changes in sparsity, sample size, noise level, and predictor correlation affect the relative performance of the competing criteria. In the sparser and more challenging setting, Stability Selection and EBIC generally showed higher T.N but lower T.P, whereas doubleS-GCV achieved the highest T.P and AUROC and the lowest MSE across all coefficient-function types. These results demonstrate that doubleS-GCV maintains favorable edge-selection and estimation performance under substantially different simulation conditions.

### 2.2. Cytarabine Sensitivity-Specific Gene Network Analysis

#### 2.2.1. Evaluation

We first evaluated gene network estimation accuracy. For the 1000 target genes, we estimated the varying coefficient model in Equation (1) and then evaluated our strategy based on the MSE of the estimated expression levels for 904 cell lines. We compared the estimation accuracy of our strategy with those of other model selection criteria (AIC, BIC, AICc, and HQC), as presented in Table 4.

As shown in Table 4, doubleS-GCV consistently achieves lower MSE values than the competing criteria across the 20 cell lines. In particular, doubleS-GCV shows superior performance for the models corresponding to extremely sensitive and resistant cell lines (i.e., cell lines 1 and 20), indicating that the proposed criterion remains stable even under extreme biological conditions. Furthermore, pairwise Wilcoxon rank-sum tests confirm that the reductions in MSE obtained by doubleS-GCV are statistically significant compared with the competing model selection criteria. These results suggest that doubleS-GCV provides a more reliable model selection strategy for accurately estimating gene regulatory networks across heterogeneous cellular environments.

#### 2.2.2. Interpretation of Cytarabine Sensitivity-Specific Gene Network Analysis

We then interpret the estimated Cytarabine sensitivity-specific gene network. We first show how the structures of cell line-specific gene networks with varying Cytarabine sensitivity change. To this end, we analyzed 20 previously estimated networks. Each cell line was labeled from 1 to 20 according to the IC50 value, where cell line 1 (20) having the smallest (largest) IC50 value.

**Figure 2:**
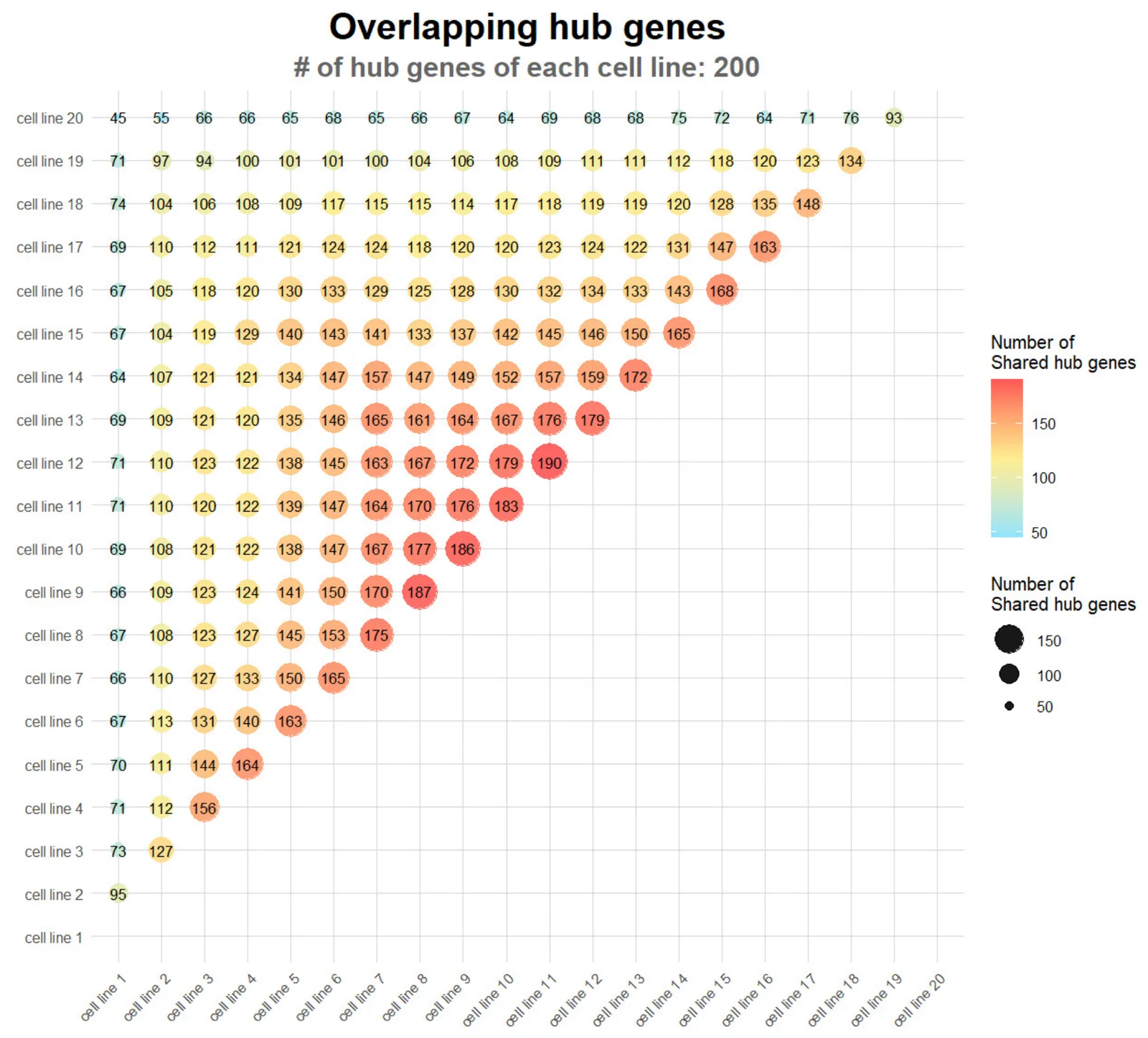
Pairwise overlap of hub genes among 20 cell line-specific gene networks. Cell lines are ordered by increasing Cytarabine half-maximal inhibitory concentration (IC_50_), from 1 (most sensitive) to 20 (most resistant). Bubble size and color indicate the number of hub genes shared by each pair of networks.

To quantify network structural similarity, we focused on the overlap of hub genes across cell lines. Hub genes play a crucial role in gene networks and serve as key indicators for identifying biological pathways or biomarkers. Therefore, the degree of hub genes overlap between networks reflects the structural similarities and provides an effective quantitative measure for comparing network structures. For each cell line–specific network, hub genes were defined as the 200 genes with the highest node degrees. Figure 2 presents a bubble plot of pairwise hub gene overlap among the 20 cell lines, illustrating how many hub genes are shared between each pair of cell lines out of these 200 selected hub genes. It shows that cell lines with similar Cytarabine sensitivity tend to share more hub genes, whereas cell lines with more distinct Cytarabine sensitivity tend to share fewer hub genes. This pattern suggests that the structures of cell line-specific gene networks reflect Cytarabine sensitivity-specific molecular characteristics.

**Figure 3:**
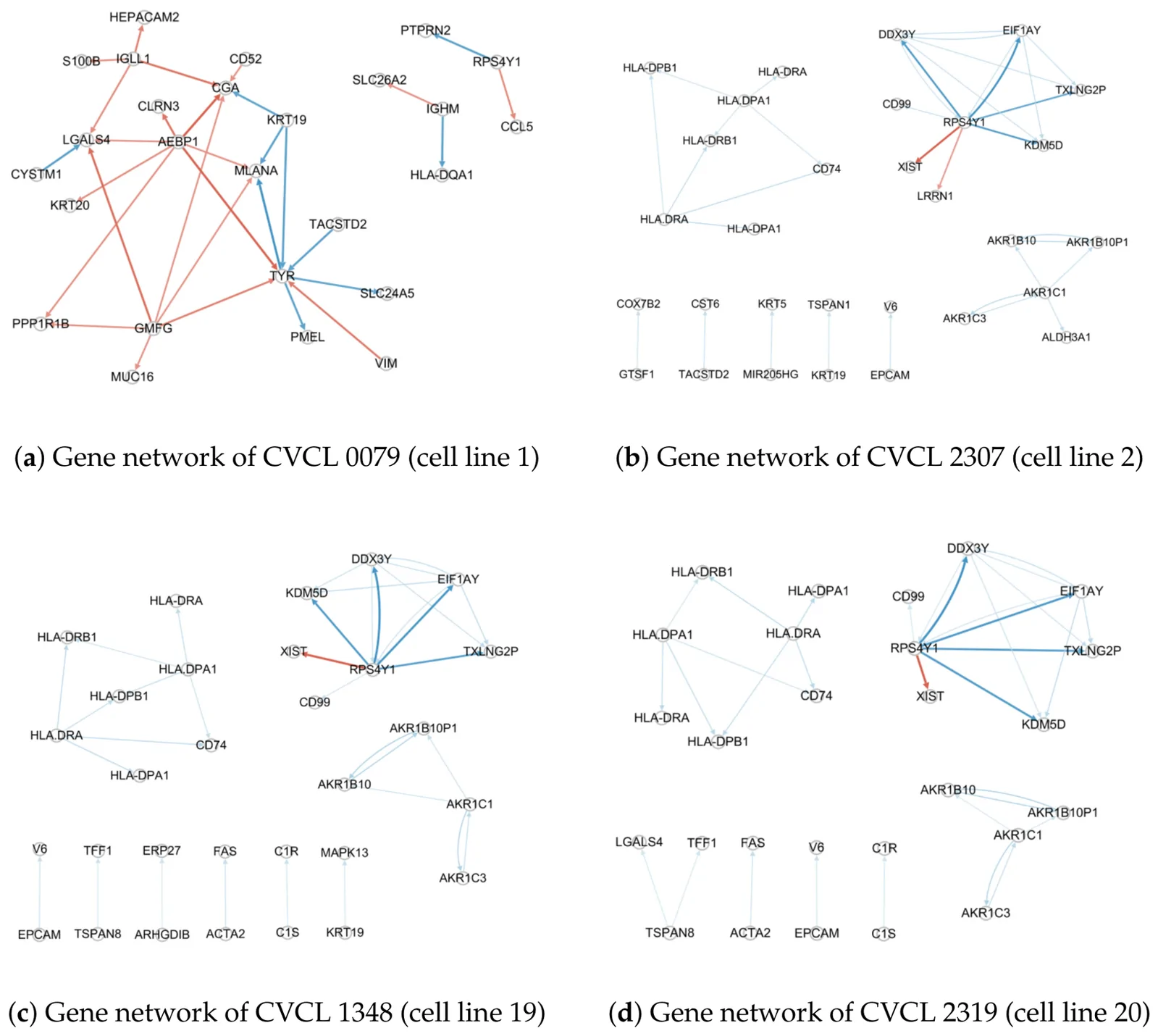
Estimated gene networks for Cytarabine-sensitive cell lines (**a**) 1 and (**b**) 2, and Cytarabine-resistant cell lines (**c**) 19 and (**d**) 20. Cell lines are numbered by increasing Cytarabine half-maximal inhibitory concentration (IC_50_). Nodes represent genes, and edges represent estimated regulatory relationships.

**Figure 4:**
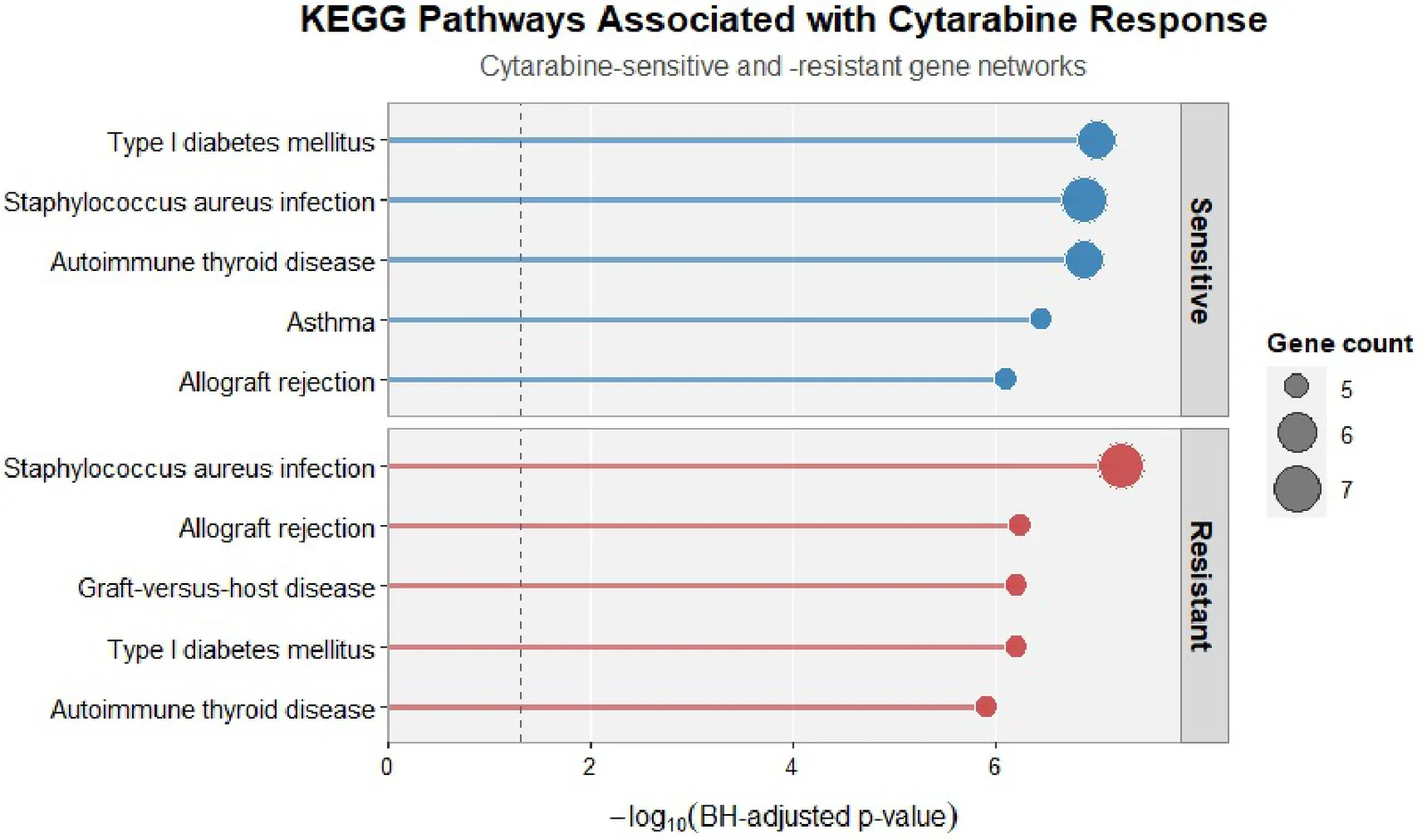
KEGG pathway enrichment analysis of genes in the Cytarabine-sensitive and -resistant networks. The five most significant pathways based on Benjamini–Hochberg-adjusted *p*-values are shown for each network. The horizontal axis represents $-\log_{10}$ of the adjusted *p*-value, point size represents the number of genes assigned to each pathway, and blue and red indicate the sensitive and resistant networks, respectively. The dashed line indicates an adjusted *p*-value of 0.05.

Figure 3 presents the estimated gene networks for the two most Cytarabine-sensitive cell lines (cell lines 1 and 2) and the two most Cytarabine-resistant cell lines (cell lines 19 and 20). We shows the KEGG pathway enrichment results in Figure 4 for the genes included in the Cytarabine-sensitive and -resistant networks shown in Figure 3. The KEGG analysis revealed a common immune-related program in both networks, including pathways associated with MHC class II-mediated antigen processing and presentation. The sensitive network additionally showed differentiation- and lineage-related features involving GMFG, AEBP1, IGLL1, TYR, and MLANA, whereas the resistant network contained an AKR1-family module involving AKR1B10, AKR1C1, and AKR1C3, suggesting metabolic and redox adaptation. Thus, the networks shared immune-regulatory characteristics but differed in their accompanying differentiation- and metabolism-related patterns. To identify Cytarabine sensitivity-specific molecular interplays, we visualized four individual cell line–specific gene networks by retaining only the top 0.005% of edges with the largest absolute edge weights, with two cell lines representing the most sensitive group and two representing the most resistant group. This allowed us to examine the unique network characteristics of each cell line before integration, as shown in Figure 3.

To support the reliability of our method, we present a list of candidate biomarker genes identified from the networks of cell lines 1 and 20, which showed the most extreme Cytarabine sensitivity, along with relevant literature references in Table 5.

Candidate biomarker genes included in the table were selected according to group-specific criteria: all identified shared genes were included, whereas sensitive-specific and resistant-specific genes were selected using a node-degree threshold of ≥4. Among the genes, RPS4Y1, LGALS4 are common to both networks, suggesting their potential as Cytarabine related biomarkers.

Resistant-specific genes, such as DDX3Y, EIF1AY, AKR1C1, HLA-DRA, and HLA-DPA1, are linked to AML through immune-related and treatment-associated biological mechanisms. DDX3Y has been reported as an immunogenic antigen expressed in male AML cells and leukemic stem cells, suggesting its potential relevance as a biomarker associated with leukemia-specific immune recognition [11]. AKR1C1 may be interpreted in the context of the AKR1C metabolic axis in AML, reflecting drug-response and differentiation-related biology rather than acting as a primary AML driver [12]. HLA-DRA and HLA-DPA1 are immune-related biomarker candidates in AML, as they are linked to MHC class II antigen presentation, immune evasion, treatment response, and clinical prognosis [13,14]. Furthermore, HLA-DPA1 was presented as an MHC class II antigen-presentation gene and was considered within the context of gene signatures predicting cytarabine (Ara-C) responsiveness, suggesting that it is an immune-related marker associated with Ara-C sensitivity rather than a direct drug-metabolism gene [15]. Sensitive-specific genes, such as TYRP1, GMFG, AEBP1, CGA, IGLL1 and MLANA, are involved in immune-associated regulation, subtype-specific genomic features, prognostic stratification, and treatment-responsive biology in AML. GMFG has been reported to be associated with both AML and cytarabine. In AML, it is linked to the disease by being significantly upregulated in LAML compared with normal tissues, showing relatively high expression compared with other cancer types, and being associated with pathways such as immune response, cytokine-cytokine receptor interaction, and hematopoietic cell lineage [17]. In relation to cytarabine, GMFG is linked through its inclusion among the top genes showing an approximately threefold increase in expression after ara-C treatment [18]. AEBP1 has been reported to be significantly upregulated in AML [19], while CGA is included in a five-gene risk model predicting AML prognosis and is therefore associated with prognosis and immune-related risk [20]. IGLL1 is linked to a subtype-specific genomic signature through its presence in the recurrently deleted 22q11.2 region in Ph(+) AML [21], and MLANA is connected to the genomic abnormality context of AML as a candidate gene related to imprinting and methylation within the 9p UPD region reported in AML [22]. It can be seen through literature that our results strongly supported by the previous studies. It implies that our strategy provide biologically reliable results for Cytarabine sensitivity-specific gene network analysis.

**Figure 5:**
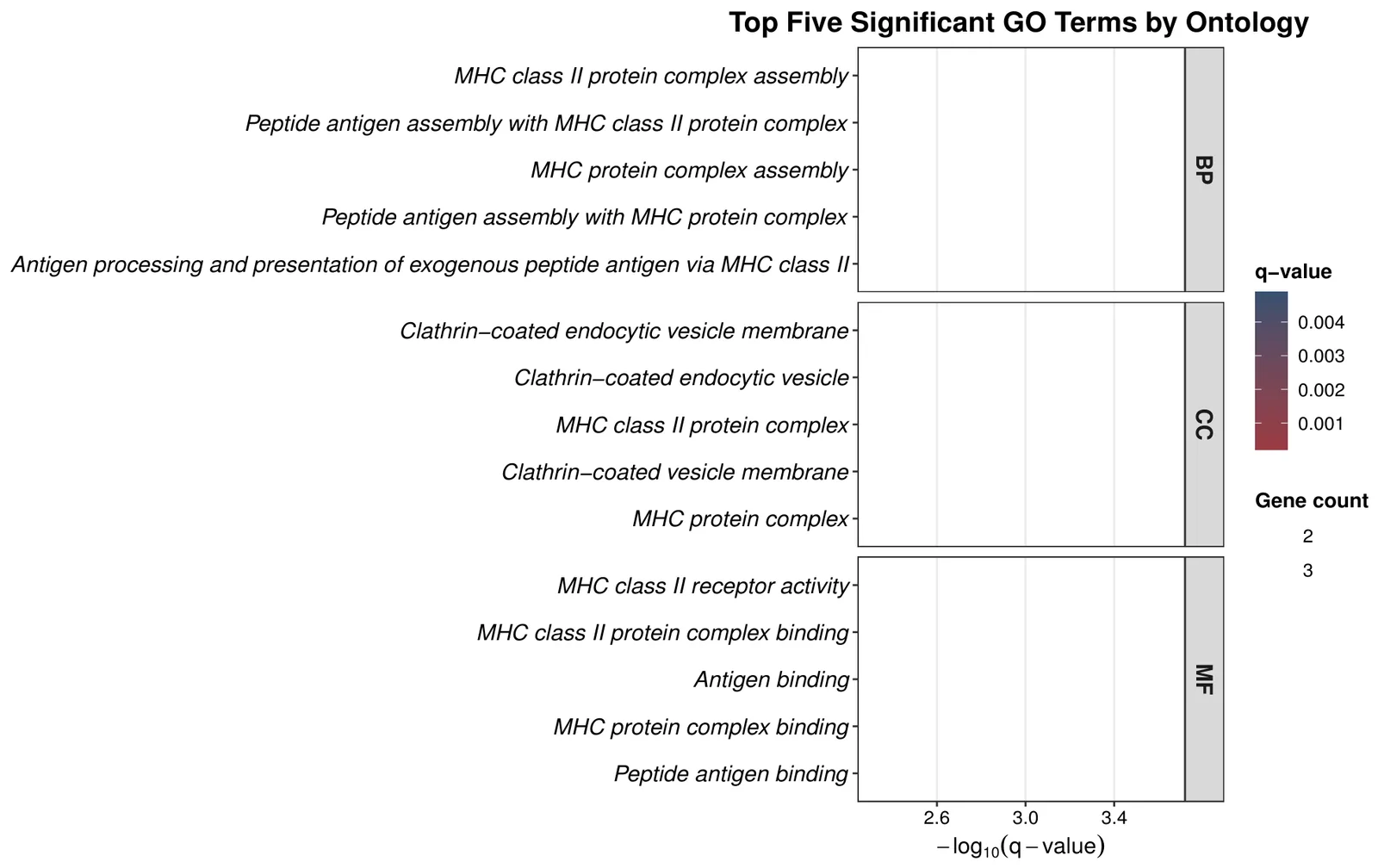
Gene Ontology enrichment analysis of the 13 unique candidate markers identified in the Cytarabine sensitivity- and resistance-specific gene networks. The five most significant terms, ranked by *q*-value, are shown for biological process (BP), cellular component (CC), and molecular function (MF). Point size represents the number of genes associated with each GO term, and color represents the corresponding *q*-value.

To investigate the biological functions associated with the identified candidate markers, we performed Gene Ontology (GO) enrichment analysis using the 13 unique genes listed in Table 5. Significant GO terms were identified using a Benjamini–Hochberg-adjusted *q*-value threshold of 0.05. As shown in Figure 5, the candidate markers were significantly enriched in biological processes related to MHC class II protein complex assembly, peptide antigen assembly, and antigen processing and presentation. The enriched cellular-component terms included MHC protein complexes and clathrin-coated endocytic vesicles and their membranes. Molecular-function enrichment was primarily associated with MHC class II receptor activity, MHC protein complex binding, and antigen and peptide antigen binding. These results provide functional context for the identified candidate markers and suggest their involvement in antigen processing and presentation and MHC class II-mediated immune functions. Thus, the enrichment analysis complements the gene-specific literature evidence presented in Table 5, although further validation using independent datasets will be required to establish their direct roles in AML and Cytarabine response.

### 2.3. Sensitivity of Network Inference to Hyperparameter Selection

To examine the sensitivity of the inferred networks to hyperparameter selection, we conducted an additional analysis comparing the networks obtained using the proposed doubleS-GCV method with those estimated under several fixed hyperparameter configurations. Specifically, two cell line–specific gene networks were estimated under two alternative settings: LASSO with small bandwidth and LASSO with large bandwidth. The comparison results are summarized in Table 6.

For each setting, we evaluated (i) the total number of edges in the inferred network, (ii) the number of overlapping edges with the doubleS-GCV network among the top 1000 edges ranked by absolute weight, and (iii) the number of overlapping hub genes (node degree ≥ 5) with those identified by the doubleS-GCV network. As shown in Table 6, the total number of inferred edges varies substantially across different hyperparameter configurations. These findings indicate that the inferred network topology can be highly sensitive to the choice of hyperparameters. The larger number of edges obtained using the kernel-based elastic net with doubleS-GCV is consistent with differences in the variable-selection properties of elastic net and lasso. When the number of candidate regulators exceeds the sample size, lasso selects at most as many predictors as the number of observations under the usual general-position conditions, whereas elastic net is not subject to this restriction and can retain groups of correlated regulators [23]. Consequently, elastic net can select more regulators for each target gene, yielding a denser network. However, network density also depends on the selected regularization strengths and kernel bandwidths, and a larger edge count does not necessarily imply greater biological accuracy. Overall, this comparison highlights the importance of appropriate hyperparameter selection in gene network inference. The proposed doubleS-GCV approach provides a data-driven and principled framework for selecting hyperparameters through the generalized cross-validation criterion, thereby reducing reliance on arbitrarily fixed parameter settings and improving the stability of the resulting network estimates.

## 3. Discussion

In this study, we proposed a sample-specific model evaluation criterion, i.e., doubleS-GCV for cell line characteristic-specific gene networks. The ordinary cross-validation approaches are computationally inefficient and provide an averaged model evaluation results across all samples. Thus, the existing CV-based analysis cannot perform well for sample-specific analysis. The developed doubleS-GCV overcomes these limitations, i.e., our strategy significantly reduces the computational burden while maintaining effective model selection performance for sample-specific analysis.

Simulation studies demonstrated that the proposed doubleS-GCV provides computationally efficient results while achieving high accuracy in gene selection and network estimation compared to existing model selection criteria (i.e., AIC, BIC, AICC, HQC). We applied doubleS-GCV to GDSC cancer cell lines to investigate Cytarabine response-associated gene networks. The estimated networks exhibited distinct interaction patterns across Cytarabine sensitivity levels, suggesting that molecular relationships vary with drug response. Candidate genes identified from the Cytarabine-sensitive and -resistant networks were supported by previous studies. However, because the analyzed cohort was not restricted to AML, these findings should be interpreted as Cytarabine response-associated patterns across cancer cell lines rather than as AML-specific mechanisms. Further validation in an independent AML-specific cohort is required to establish their relevance to AML. The Cytarabine-sensitive and -resistant gene networks show clearly distinct interaction patterns. The sensitive networks are linked to cell differentiation, while resistant networks involve genes related to proliferation and drug resistance. We expect that our methodology could be a useful tool for sample-specific gene network analysis and other forms of sample-specific analysis.

In terms of its methodological properties, doubleS-GCV approximates sample-specific leave-one-out prediction error using the hat matrix derived through local quadratic approximation, thereby balancing model fit and effective complexity without repeated model fitting. It is expected to be particularly advantageous when regulatory relationships vary across samples and conventional cross-validation is computationally prohibitive or insufficiently sensitive to local model performance. However, its performance depends on the suitability of the varying coefficient model, kernel-based local weighting, the local quadratic approximation, and the candidate hyperparameter grid. Stable estimation also requires a sufficient local effective sample size around each target sample. Moreover, doubleS-GCV evaluates models generated by an underlying network estimation procedure and therefore does not overcome limitations inherent in that estimator. Although favorable empirical performance was observed, formal asymptotic properties, including selection consistency and oracle properties, remain to be established in future work.

This study has two main limitations. First, although doubleS-GCV reduces the number of repeated model evaluations required by conventional cross-validation, the underlying network estimation procedure may remain computationally demanding for networks involving thousands of genes. In particular, estimating sample-specific networks across numerous target samples and an extensive hyperparameter grid requires substantial computational resources. Further developments, including parallel implementation, variable-screening procedures, and scalable network estimation algorithms, will therefore be necessary for genome-wide applications. Second, the candidate genes identified in the Cytarabine-sensitive and Cytarabine-resistant networks were supported by previous studies but were not subjected to direct biological or experimental validation. Because the GDSC analysis included cell lines from multiple cancer types, further validation in an independent AML-specific cohort is required. Future studies should compare the inferred networks with established AML regulatory networks and validate the identified genes and regulatory relationships using independent datasets, patient-derived samples, and functional experiments to establish their biological and clinical relevance.

## 4. Materials and Methods

Let *n* denote the number of cell lines, and let ${\left\{\left(t_{i\ell },r_{ij},m_{i}\right)\right\}}_{i=1}^{n}$ represent the observed data, where the *i*-th cell line is treated as the *i*-th statistical sample. Here, $t_{i\ell }$ denotes the expression level of the *ℓ*-th target gene in the *i*-th cell line, $r_{ij}$ denotes the expression level of the *j*-th regulator gene in the *i*-th cell line, and $m_{i}$ represents the corresponding modulator value of the *i*-th cell line. Throughout the manuscript, the index *i* consistently refers to the cell line (i.e., a single statistical sample) index. We denote α as the index of the target sample (i.e., target cell line) in the cell line–specific gene network inference. For clarity, the major symbols used in the model formulation and derivation of doubleS-GCV are summarized in Table A1 in Appendix B.

Suppose $r_{i1},...,r_{iq}$ are *q* possible regulators that may control the transcription of the $\ell^{\mathrm{th}}$ target gene $t_{i\ell }$. The gene network for the $\ell^{\mathrm{th}}$ target gene can be estimated by the following linear regression model:

$$t_{i\ell }=\sum_{j=1}^{q}\beta_{j\ell }\cdot r_{ij}+\epsilon_{i\ell },$$

where $\beta_{j\ell }$ is the coefficient that quantifies the strength of the $j^{\mathrm{th}}$ regulator gene toward the $\ell^{\mathrm{th}}$ target gene. The error term $\epsilon_{i\ell }$ is assumed to follow a normal distribution $N(0,\sigma^{2})$. The primary goal is to estimate the regression coefficient $\beta_{\ell }$ that represents the edge weights toward $\ell^{\mathrm{th}}$ target gene in the gene network. To estimate the gene network (i.e., $\beta_{\ell }$), the following elastic net has often been used [23]

$$L\left(\beta_{\ell }\right)=\frac{1}{2}\sum_{i=1}^{n}{\left\{t_{i\ell }-\sum_{j=1}^{q}\beta_{j\ell }\cdot r_{ij}\right\}}^{2}+P_{\gamma_{\ell },\delta_{\ell }}\left(\beta_{\ell }\right),$$

where

$$P_{\gamma_{\ell },\delta_{\ell }}\left(\beta_{\ell }\right)=\gamma_{\ell }\sum_{j=1}^{q}\left[\frac{1}{2}\left(1-\delta_{\ell }\right)\beta_{j\ell }^{2}+\delta_{\ell }\left|\beta_{j\ell }\right|\right],$$

where $\gamma_{\ell }$ and $\delta_{\ell }$ are hyperparameters that control the overall strength of regularization and the balance between $L_{1}$ and $L_{2}$ -norm penalties, respectively. Although the elastic net is widely used for gene network estimation, it produces a single network inferred from all samples. Consequently, the estimated network reflects aggregate molecular interplays across the *n* samples and does not allow for the inference of cell line–specific molecular interactions.

Thus, we cannot reveal the sample-specific molecular interplays by using the ordinary $L_{1}$-type regularization methods.

### 4.1. Sample-Specific Gene Network Estimation

To settle on the issue and effectively estimate cell line characteristic-specific gene network of the $\alpha^{th}$ sample, we considered the following varying coefficient model [4]

(1) $$t_{\alpha \ell }=\sum_{j=1}^{q}\beta_{j\ell }\left(M=m_{\alpha }\right)\cdot r_{\alpha j}+\epsilon_{\alpha \ell },$$

where $m_{\alpha }$ denotes the modulator value of the $\alpha^{\mathrm{th}}$ cell line. It represents a continuous sample-specific characteristic that defines the biological condition under which the gene network is estimated, such as drug sensitivity. For example, in the GDSC cancer cell-line analysis, $m_{\alpha }$ corresponds to the IC50 value of Cytarabine for the $\alpha^{th}$ cell line. In cell line–specific gene network inference, the modulator values determine the local weighting structure in the Gaussian kernel and enable systematic comparison of gene network structures across varying biological conditions (see Equation (2)). From a statistical perspective, the modulator value serves as an effect-modifying variable in the varying coefficient model, allowing the regression coefficients to vary smoothly along a continuous biological gradient.

The varying coefficient vector $\beta_{\ell }\left(M=m_{\alpha }\right)$ quantifies the regulatory effect on $\ell^{\mathrm{th}}$ target gene, specific to the modulator value of the $\alpha^{\mathrm{th}}$ sample. The cell line characteristic-specific gene network for the $\alpha^{th}$ cell line can be estimated by the following kernel-based $L_{1}$-type regularization method [4],

(2) $$L\left(\beta_{\ell \alpha }|h_{\ell \alpha }\right)=\frac{1}{2}\sum_{i=1}^{n}{\left\{t_{i\ell }-\sum_{j=1}^{q}\beta_{j\ell \alpha }\cdot r_{ij}\right\}}^{2}K\left(m_{i}-m_{\alpha }|h_{\ell \alpha }\right)+P_{\gamma_{\ell \alpha },\delta_{\ell \alpha }}\left(\beta_{\ell \alpha }\right),$$

where $\beta_{j\ell \alpha }=\beta_{j\ell }\left(M=m_{\alpha }\right)$ and

$$P_{\gamma_{\ell \alpha },\delta_{\ell \alpha }}\left(\beta_{\ell \alpha }\right)=\gamma_{\ell \alpha }\sum_{j=1}^{q}\left[\frac{1}{2}\left(1-\delta_{\ell \alpha }\right)\beta_{j\ell \alpha }^{2}+\delta_{\ell \alpha }\left|\beta_{j\ell \alpha }\right|\right],$$

$$K\left(m_{i}-m_{\alpha }|h_{\ell \alpha }\right)=\exp\left\{-\frac{{\left(m_{i}-m_{\alpha }\right)}^{2}}{h_{\ell \alpha }}\right\}.$$

The Gaussian kernel function $K(m_{i}-m_{\alpha }|h_{\ell \alpha })$ assigns weights to each cell line for gene network estimation of the $\alpha^{\mathrm{th}}$ sample. When estimating the model for the $\alpha^{\mathrm{th}}$ cell line, neighboring cell lines with similar modulator values receive higher weights, whereas those with distant values receive lower weights. This enables the network of the $\alpha^{\mathrm{th}}$ cell line to be modeled primarily based on biologically similar neighbors. The hyperparameter $h_{\ell \alpha }$ controls the kernel bandwidth and determines the range of neighboring cell lines.

### 4.2. Existing Methods for Model Evaluation

The estimation results of cell line-specific gene networks heavily rely on the hyperparameters $\delta_{\ell \alpha },\gamma_{\ell \alpha }$ and $h_{\ell \alpha }$. Thus, it is important to choose them appropriately.

#### 4.2.1. Information Theoretic Criteria

Information-theoretic criteria have long been established as standard tools for model evaluation and hyperparameter selection.

- Akaike Information Criterion (AIC, [24]):
  (3) $$\begin{array}{c} \mathrm{AIC}=\frac{\left|\right|t_{\ell }-{\hat{t}}_{\ell }{\left|\right|}^{2}}{n\sigma^{2}}+\frac{2}{n}\hat{df}, \end{array}$$
  where $\hat{df}$ is the degree of freedom of the estimated model.
- Bayesian Information Criterion (BIC, [24]):
  (4) $$\begin{array}{c} \mathrm{BIC}=\frac{\left|\right|t_{\ell }-{\hat{t}}_{\ell }{\left|\right|}^{2}}{n\sigma^{2}}+\frac{\log(n)}{n}\hat{df}. \end{array}$$
- Hannah and Quinn Criterion (HQC, [25]):
  (5) $$\begin{array}{c} \mathrm{HQC}=\frac{\left|\right|t_{\ell }-{\hat{t}}_{\ell }{\left|\right|}^{2}}{n\sigma^{2}}+\frac{2\log\{\log(n)\}}{n}\hat{df}. \end{array}$$
- AIC with a sample correction (AICc, [26]):
  (6) $$\begin{array}{c} \mathrm{AICc}=AIC+\frac{2\hat{df}\left(\hat{df}+1\right)}{n^{2}-\hat{df}-n} \end{array}$$

#### 4.2.2. Cross-Validation

One of the most commonly used techniques for hyperparameters selection is Cross-validation (CV) and the CV error of model for the $\ell^{\mathrm{th}}$ target gene is as follows:

(7) $${\mathrm{CV}}_{\ell }=\frac{1}{n}\sum_{\alpha =1}^{n}{\left\{t_{\alpha \ell }-\sum_{j=1}^{q}{\hat{\beta }}_{j\ell \alpha }^{\left(-\alpha \right)}\cdot r_{\alpha j}\right\}}^{2},$$

where the coefficient ${\hat{\beta }}_{j\ell \alpha }^{\left(-\alpha \right)}$ is estimated by using n−1 samples, obtained by removing the $\alpha^{\mathrm{th}}$ data point from *n* observations.

While the CV in Equation (7) provides an intuitive measure of predictive performance, it has two critical limitations. First, CV imposes a substantial computational burden because the model must be re-estimated *n* times, each time leaving out one observation. In particular, leave-one-out cross-validation (LOO-CV) requires *n* separate model fittings, which becomes computationally prohibitive in high-dimensional gene network analysis. This issue is further amplified in cell line–specific gene network analysis, where a separate model is constructed for each of the *n* cell lines. For each target cell line indexed by α (α=1,…,n), model evaluation via LOO-CV again requires leaving out each observation in turn. Consequently, a total of n×n model estimations are required for cell line–specific gene network inference with *n* cell lines. Second, the ordinary CV provides an averaged model evaluation across all samples, as the prediction error is computed by aggregating residuals over all cell lines. In other words, CV selects hyperparameters that optimize global predictive performance rather than the performance for a specific target cell line. However, our objective is to infer cell line–specific molecular interplays, where each target cell line may require distinct model complexity. Therefore, the averaged nature of CV fails to directly reflect the performance of a sample-specific model. As a result, the standard CV is not well suited for evaluating cell line characteristic-specific gene network models.

### 4.3. Model Evaluation Criterion for Cell Line-Specific Model

In this study, we propose a model selection criterion, called sample-specific Generalized Cross-Validation (ss-GCV), i.e., doubleS-GCV, for sample-specific gene network analysis. In order to derive doubleS-GCV, we first point out the objective function of the kernel-based $L_{1}$-type regularized regression model in Equation (2) is reformulated without the kernel function as follows:

(8) $$\begin{array}{cc} L(\beta_{l\alpha }|h_{\ell \alpha })\; & =\frac{1}{2}\sum_{i=1}^{n}{\left\{t_{i\ell }-\sum_{j=1}^{q}\beta_{j\ell \alpha }\cdot r_{ij}\right\}}^{2}K\left(m_{i}-m_{\alpha }|h_{\ell \alpha }\right)+P_{\gamma_{\ell \alpha },\delta_{\ell \alpha }}\left(\beta_{j\ell \alpha }\right)\\ \; & =\frac{1}{2}{\left(t_{\ell }-R\beta_{\ell \alpha }\right)}^{T}K_{\ell \alpha }\left(t_{\ell }-R\beta_{\ell \alpha }\right)+P_{\gamma_{\ell \alpha },\delta_{\ell \alpha }}\left(\beta_{\ell \alpha }\right)\\ \; & =\frac{1}{2}{\left(t_{\ell \alpha }^{*}-R_{\ell \alpha }^{*}\beta_{\ell \alpha }\right)}^{T}\left(t_{\ell \alpha }^{*}-R_{\ell \alpha }^{*}\beta_{\ell \alpha }\right)+P_{\gamma_{\ell \alpha },\delta_{\ell \alpha }}\left(\beta_{\ell \alpha }\right), \end{array}$$

where

$$K_{\ell \alpha }=\mathrm{diag}\{K\left(m_{1}-m_{\alpha }|h_{\ell \alpha }\right),\cdots ,K\left(m_{n}-m_{\alpha }|h_{\ell \alpha }\right)\},$$

$$t_{\ell \alpha }^{*}=\left(\begin{array}{c} \sqrt{K(m_{1}-m_{\alpha }|h_{\ell \alpha })}\cdot t_{1\ell }\\ \vdots \\ \sqrt{K(m_{n}-m_{\alpha }|h_{\ell \alpha })}\cdot t_{n\ell } \end{array}\right),$$

$$R_{\ell \alpha }^{*}=\left(\begin{array}{ccc} \sqrt{K(m_{1}-m_{\alpha }|h_{\ell \alpha })}\cdot r_{11} & \cdots & \sqrt{K(m_{1}-m_{\alpha }|h_{\ell \alpha })}\cdot r_{1q}\\ \vdots & \ddots & \vdots \\ \sqrt{K(m_{1}-m_{\alpha }|h_{\ell \alpha })}\cdot r_{n1} & \cdots & \sqrt{K(m_{1}-m_{\alpha }|h_{\ell \alpha })}\cdot r_{nq} \end{array}\right).$$

Based on the reformulated objective function in Equation (8), the cross-validation error of the model for the $\ell^{\mathrm{th}}$ target gene and the $\alpha^{\mathrm{th}}$ target cell line is defined as follows:

(9) $${\mathrm{CV}}_{\ell \alpha }=\frac{1}{n}\sum_{i=1}^{n}{\left\{t_{i\ell \alpha }^{*}-\sum_{j=1}^{q}{\hat{\beta }}_{j\ell \alpha }^{\left(-i\right)}\cdot r_{ij\ell \alpha }^{*}\right\}}^{2}.$$

To derive the generalized cross-validation (GCV) criterion, it is essential to compute the hat matrix. However, the hat matrix cannot be computed in closed form, because the $L_{1}$-type penalty term is not differentiable. To address this limitation, we employ the local quadratic approximation (LQA) of the $L_{1}$-type penalty [9], with technical details provided in Appendix A. Using this approximation, the elastic net penalty term can be reformulated as follows,

(10) $$\begin{array}{cc} P_{\gamma_{l\alpha },\delta_{l\alpha }}\left(\beta_{l\alpha }\right)\; & =\gamma_{l\alpha }\sum_{j=1}^{q}\left[\frac{1}{2}\left(1-\delta_{l\alpha }\right)\beta_{j\ell \alpha }^{2}+\delta_{l\alpha }\left|\beta_{j\ell \alpha }\right|\right]\\ \; & \approx \gamma_{l\alpha }\sum_{j=1}^{q}\left[\frac{1}{2}\left(1-\delta_{l\alpha }\right)\beta_{j\ell \alpha }^{2}+\delta_{l\alpha }\left|\beta_{j\ell \alpha 0}\right|+\frac{1}{2}\left\{\frac{\delta_{l\alpha }}{|\beta_{j\ell \alpha 0}|}\right\}\left(\beta_{j\ell \alpha }^{2}-\beta_{j\ell \alpha 0}^{2}\right)\right]\\ \; & =\frac{\gamma_{l\alpha }}{2}\sum_{j=1}^{q}\left[\left(1-\delta_{l\alpha }\right)+\frac{\delta_{l\alpha }}{|\beta_{j\ell \alpha 0}|}\right]\beta_{j\ell \alpha }^{2}+\gamma_{l\alpha }\sum_{j=1}^{q}\left[\delta_{l\alpha }\left|\beta_{j\ell \alpha 0}\right|-\frac{\delta_{l\alpha }}{2|\beta_{j\ell \alpha 0}|}\beta_{j\ell \alpha 0}^{2}\right]. \end{array}$$

Thus, the hat matrix of the kernel-based $L_{1}$-type regularized regression model in Equation (8) can be given as follows:

(11) $$H_{\ell \alpha }=R_{\ell \alpha }^{*}{\left(R_{\ell \alpha }^{{*}^{T}}R_{\ell \alpha }^{*}+\Sigma_{\gamma_{\ell \alpha },\delta_{\ell \alpha }}\left({\hat{\beta }}_{\ell \alpha }\right)\right)}^{-1}R_{\ell \alpha }^{{*}^{T}},$$

where

$$\Sigma_{\gamma_{\ell \alpha },\delta_{\ell \alpha }}\left({\hat{\beta }}_{\ell \alpha }\right)=\gamma_{\ell \alpha }\mathrm{diag}\left\{\left[\left(1-\delta_{\ell \alpha }\right)+\frac{\delta_{\ell \alpha }}{|\beta_{1\ell \alpha 0}|}\right],\cdots ,\left[\left(1-\delta_{\ell \alpha }\right)+\frac{\delta_{\ell \alpha }}{|\beta_{q\ell \alpha 0}|}\right]\right\}.$$

We then derive doubleS-GCV based on the reformulated objective function in Equation (8) and the hat matrix in Equation (11). In line with [6], we derive GCV from the CV in Equation (9).

We first define a vector z as follows:

$$z={\left(t_{1\ell \alpha }^{*},\cdots ,\sum_{j=1}^{q}{\hat{\beta }}_{j\ell \alpha }^{\left(-i\right)}\cdot r_{ij\ell \alpha }^{*},\cdots ,t_{n\ell \alpha }^{*}\right)}^{T}.$$

The fact that ${\hat{\beta }}_{\ell \alpha }^{\left(-i\right)}$ minimizes

$$f\left(\beta_{\ell \alpha }\right)=\frac{1}{2}\sum_{k=1}^{n}{\left\{z_{k}-\sum_{j=1}^{q}\beta_{j\ell \alpha }\cdot r_{kj\ell \alpha }^{*}\right\}}^{2}+\frac{1}{2}\begin{array}{c} \sum_{j=1}^{q} \end{array}P_{\gamma_{\ell \alpha },\delta_{\ell \alpha }}\left(\beta_{j\ell \alpha }\right)$$

can be demonstrated based on the following inequality:

$$\begin{array}{cc} f(\beta_{\ell \alpha })\; & \geq \frac{1}{2}\sum_{k\neq i}^{n}{\left\{z_{k}-\sum_{j=1}^{q}\beta_{j\ell \alpha }\cdot r_{kj\ell \alpha }^{*}\right\}}^{2}+\frac{1}{2}\begin{array}{c} \sum_{j=1}^{q} \end{array}P_{\gamma_{\ell \alpha },\delta_{\ell \alpha }}\left(\beta_{j\ell \alpha }\right)\\ \; & \geq \frac{1}{2}\sum_{k\neq i}^{n}{\left\{z_{k}-\sum_{j=1}^{q}{\hat{\beta }}_{j\ell \alpha }^{\left(-i\right)}\cdot r_{kj\ell \alpha }^{*}\right\}}^{2}+\frac{1}{2}\begin{array}{c} \sum_{j=1}^{q} \end{array}P_{\gamma_{\ell \alpha },\delta_{\ell \alpha }}\left({\hat{\beta }}_{j\ell \alpha }^{\left(-i\right)}\right)\\ \; & =\frac{1}{2}\sum_{k=1}^{n}{\left\{z_{k}-\sum_{j=1}^{q}{\hat{\beta }}_{j\ell \alpha }^{\left(-i\right)}\cdot r_{kj\ell \alpha }^{*}\right\}}^{2}+\frac{1}{2}\begin{array}{c} \sum_{j=1}^{q} \end{array}P_{\gamma_{\ell \alpha },\delta_{\ell \alpha }}\left({\hat{\beta }}_{j\ell \alpha }^{\left(-i\right)}\right). \end{array}$$

Thus, we obtain:

$$\sum_{j=1}^{q}{\hat{\beta }}_{j\ell \alpha }^{\left(-i\right)}\cdot r_{ij\ell \alpha }^{*}=\sum_{k=1}^{n}h_{ik}\cdot z_{k},$$

where $h_{ik}$ is the ${\left(i,k\right)}^{th}$ component of the hat matrix $H_{l\alpha }$. From this result, we obtain the following formula:

$$\begin{array}{cc} \sum_{j=1}^{q}{\hat{\beta }}_{j\ell \alpha }^{\left(-i\right)}\cdot r_{ij\ell \alpha }^{*}-t_{i\ell \alpha }^{*}\; & =\sum_{k=1}^{n}h_{ik}\cdot z_{k}-t_{i\ell \alpha }^{*}\\ \; & =\sum_{k\neq i}^{n}h_{ik}\cdot t_{k\ell \alpha }^{*}-t_{i\ell \alpha }^{*}+h_{ii}\sum_{j=1}^{q}{\hat{\beta }}_{j\ell \alpha }^{\left(-i\right)}\cdot r_{ij\ell \alpha }^{*}\\ \; & =\sum_{k=1}^{n}h_{ik}\cdot t_{k\ell \alpha }^{*}-t_{i\ell \alpha }^{*}+h_{ii}\left\{\sum_{j=1}^{q}{\hat{\beta }}_{j\ell \alpha }^{\left(-i\right)}\cdot r_{ij\ell \alpha }^{*}-t_{i\ell \alpha }^{*}\right\}\\ \; & =\sum_{j=1}^{q}{\hat{\beta }}_{j\ell \alpha }\cdot r_{ij\ell \alpha }^{*}-t_{i\ell \alpha }^{*}+h_{ii}\left\{\sum_{j=1}^{q}{\hat{\beta }}_{j\ell \alpha }^{\left(-i\right)}\cdot r_{ij\ell \alpha }^{*}-t_{i\ell \alpha }^{*}\right\}, \end{array}$$

and thus

$$t_{i\ell \alpha }^{*}-\sum_{j=1}^{q}{\hat{\beta }}_{j\ell \alpha }^{\left(-i\right)}\cdot r_{ij\ell \alpha }^{*}=\frac{t_{i\ell \alpha }^{*}-\sum_{j=1}^{q}{\hat{\beta }}_{j\ell \alpha }\cdot r_{ij\ell \alpha }^{*}}{1-h_{ii}}.$$

Consequently, the CV in Equation (9) can be given as:

$$\begin{array}{cc} {\mathrm{CV}}_{\ell \alpha }\; & =\frac{1}{n}\sum_{i=1}^{n}{\left\{t_{i\ell \alpha }^{*}-\sum_{j=1}^{q}{\hat{\beta }}_{j\ell \alpha }^{\left(-i\right)}\cdot r_{ij\ell \alpha }^{*}\right\}}^{2}\\ \; & =\frac{1}{n}\sum_{i=1}^{n}{\left\{\frac{t_{i\ell \alpha }^{*}-\sum_{j=1}^{q}{\hat{\beta }}_{j\ell \alpha }\cdot r_{ij\ell \alpha }^{*}}{1-h_{ii}}\right\}}^{2}. \end{array}$$

It implies that the CV criterion based on *n* estimations can be computed without repeated estimations. By replacing $1-h_{ii}$ to its average $1-\frac{1}{n}\mathrm{tr}\left(H_{\ell \alpha }\right)$, we propose sample-specific GCV as follows:

$${\mathrm{doubleS-GCV}}_{\ell \alpha }=\frac{1}{n}\frac{\sum_{i=1}^{n}{\left\{t_{i\ell \alpha }^{*}-\sum_{j=1}^{q}{\hat{\beta }}_{j\ell \alpha }\cdot r_{ij\ell \alpha }^{*}\right\}}^{2}}{{\left\{1-\frac{1}{n}\mathrm{tr}\left(H_{\ell \alpha }\right)\right\}}^{2}},$$

The proposed doubleS-GCV significantly reduces the computational burden while maintaining effective model selection performance. Furthermore, our strategy provides sample-specific model evaluation results.

### 4.4. Simulation Study Design

#### 4.4.1. Evaluation 1: Computational Efficiency

To demonstrate the computational efficiency of doubleS-GCV, we generated simulation datasets based on the following varying coefficient model:

(12) $$t_{\alpha }=\sum_{j=1}^{q}\beta_{j\alpha }\cdot r_{\alpha j}+\epsilon_{\alpha },\;\alpha =1,\cdots ,n,$$

where $\beta_{j\alpha }=\beta_{j}\left(M=m_{\alpha }\right)$. $r_{i}$ is a *q*-dimensional predictor vector, and the error terms $\epsilon_{\alpha }$ follow a standard normal distribution. The correlation of $r_{\alpha j}$ and $r_{\alpha k}$ is $0.5^{\left|j-k\right|}$ for *q*-dimensional multivariate normal distribution with zero mean. The modulator values $\left(m_{1},\cdots ,m_{n}\right)$ are generated from a uniform distribution, U(0,1). We considered four values of the number of regulator genes, q∈{50,200,500,1000}, and two sample sizes, n∈{25,100}. Among the *q* regulators, 20% of *q* genes were randomly selected as crucial variables. For the crucial variables, the varying coefficient value decreased linearly from 3 to 1 as the modulator value $m_{\alpha }$ increased. The remaining 80% of the variables were treated as noise variables with zero coefficients.

#### 4.4.2. Evaluation 2: Accuracy in Gene Network Estimation

**Figure 6:**
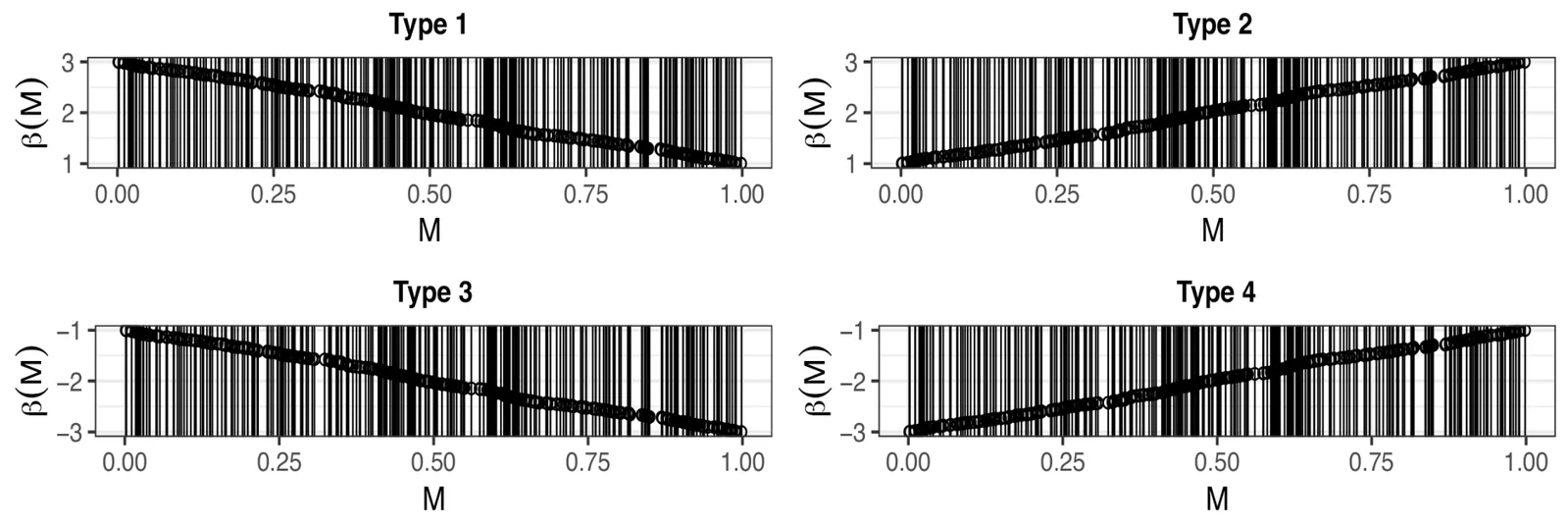
Four types of varying coefficient functions β(M) for the crucial variables considered in the simulation study. The horizontal axis represents the modulator value M∈[0,1] for *n* cell lines, and the vertical axis represents the corresponding coefficient value β(M). Type 1 (Type 3) exhibits monotonically decreasing coefficients β(M) from 3 to 1 (from −1 to −3) as *M* increases, whereas Type 2 (Type 4) exhibits monotonically increasing coefficients from 1 to 3 (from −3 to −1). Types 1 and 2 correspond to positive coefficients, while Types 3 and 4 correspond to negative coefficients.

In order to evaluate the accuracy of doubleS-GCV in terms of edge selection and edge weight estimation, we considered the varying coefficient model in Equation (12) for n=300 and q=500, where 100 genes are crucial regulators. For the crucial variables, we considered four different types of varying coefficient functions, as shown in Figure 6. The regularization parameter was evaluated using three equally spaced values between 0.1 and 0.5, the elastic-net mixing parameter using three equally spaced values between 0.1 and 0.9, and the Gaussian-kernel bandwidth using three equally spaced values between 0.1 and 3. Thus, a total of 3×3×3=27 hyperparameter combinations were evaluated for each target sample. All functions change with respect to the modulator value, but differ in direction and coefficient range. To assess edge selection accuracy, we computed the true positive (T.P) rate, defined as the proportion of correctly selected crucial variables, the true negative (T.N) rate, defined as the proportion of correctly identified irrelevant variables. We also compute mean squared error to evaluate the edge weight estimation accuracy. Additionally, the area under the receiver operating characteristic (ROC) curve (AUROC) was used as a summary measure of edge selection performance. For each simulation dataset, the AUROC was computed separately for each sample and then averaged, resulting in one averaged AUROC value per simulation.

#### 4.4.3. Evaluation 3: Performance Under a Challenging Simulation Scenario

To further evaluate the performance of doubleS-GCV under a more challenging setting, we considered the varying coefficient model in Equation (12) with n=100 and q=100. Among the 100 potential regulators, 3% were randomly designated as crucial variables, while the remaining variables had zero coefficients. For the crucial variables, we used the Type 1 varying coefficient function shown in Figure 6. To introduce correlation among the predictors, the 100 variables were divided into five blocks of equal size, with 20 variables in each block. Variables within the same block had a pairwise correlation of 0.5, whereas variables belonging to different blocks were independent. The predictor vector was generated from a multivariate normal distribution according to this block-correlation structure. Independent Gaussian errors with mean zero and $\sigma^{2}=9$ were added to the response. Thus, compared with the original simulation setting, this scenario simultaneously involved a smaller sample size, a sparser coefficient structure, a higher noise level, and correlated predictors. A total of 100 independently generated datasets were evaluated.

### 4.5. Cytarabine Response Data and Experimental Setup

We applied our methodology to the Genomics of Drug Sensitivity in Cancer (GDSC) dataset, available at https://www.cancerrxgene.org (accessed on 1 January 2024), which is the largest public resource providing drug sensitivity data for cancer cells and molecular markers of drug response. The GDSC dataset includes gene expression profiles for 1018 cell lines and drug sensitivity for 970 cell lines across 402 drugs, quantified using IC50 values (i.e., the drug concentration required to inhibit cell growth by 50%; lower IC50 indicates higher sensitivity). We focused on Cytarabine, an FDA-approved drug for acute myeloid leukemia (AML). RMA-normalized basal gene expression profiles and GDSC1 fitted dose-response data were matched using COSMIC IDs, resulting in 942 matched cell lines. Among these, 904 cell lines had Cytarabine response measurements and were included in the analysis. Of the 904 analyzed cell lines, 28 were derived from AML. No missing values were imputed; only cell lines with both gene expression and Cytarabine response data were retained. The GDSC-provided LN(IC50) values were used without further transformation or dichotomization. We selected the 1000 genes with the highest expression variance. For each gene, we treated it as the target gene and used the remaining 999 genes as potential regulators when constructing Cytarabine response-associated gene networks. All 904 cell lines were ordered by their Cytarabine half-maximal inhibitory concentration (IC50) values, and sample-specific networks were estimated for 20 representative cell lines corresponding to equally spaced percentiles of the IC50 distribution (0%, 5.26%, …, 100%). The representative cell lines were selected irrespective of cancer type and therefore were not restricted to AML. This design enabled us to examine changes in gene network structure across the full range of Cytarabine sensitivity among the analyzed GDSC cancer cell lines.

For each target gene and representative cell line, the regularization parameter was evaluated using three equally spaced values between 0.1 and 0.5, the elastic-net mixing parameter using three equally spaced values between 0.1 and 0.9, and the Gaussian-kernel bandwidth using three equally spaced values between 0.1 and 3. The optimal combination was selected by minimizing doubleS-GCV over the resulting 27 candidate combinations. No iterative convergence criterion was required because all candidate combinations in the prespecified finite grid were evaluated directly. All preprocessing, gene filtering, percentile-based cell-line selection, and hyperparameter evaluation procedures were deterministic; therefore, no random seed was required for the GDSC application.

## Figures and Tables

**Table 1:** Computation time (seconds) for gene network estimation using doubleS-GCV, Akaike information criterion (AIC), Bayesian information criterion (BIC), leave-one-out cross-validation (LOOCV), and 10-fold cross-validation. Here, *q* is the number of genes and *n* is the number of cell lines. Lower values indicate greater computational efficiency.

*n*	Methods	q=50	q=200	q=500	q=1000
n=25	**doubleS-GCV**	4.082	8.148	7.875	11.141
	AIC	2.283	5.439	4.548	7.628
	BIC	2.218	5.348	5.161	7.816
	LOOCV	65.831	144.412	124.163	185.171
	10-fold CV	30.358	65.119	80.775	78.897
n=100	**doubleS-GCV**	19.045	46.239	53.708	184.042
	AIC	9.035	28.226	34.233	111.311
	BIC	9.663	28.428	33.437	107.648
	LOOCV	1591.461	5898.562	3263.468	9442.917
	10-fold CV	299.328	718.241	511.345	1328.576

**Table 2:** Comparison of gene network estimation accuracy across four types of varying coefficient functions. The table reports the true positive rate (T.P), true negative rate (T.N), their average (Avg), mean squared error (MSE), and area under the receiver operating characteristic curve (AUROC). Avg is reported as supplementary information, whereas AUROC serves as a summary measure of variable-selection performance. MSE measures estimation accuracy. The comparison includes conventional information-theoretic criteria (AIC, BIC, AICc, and HQC) as well as EBIC, EHQC, and Stability Selection. All results are averaged over 100 simulated datasets for each coefficient-function type. Bold values indicate the best performance for each metric within each function type.

Method	Type 1					Type 2				
	T.P	T.N	Avg	MSE	AUROC	T.P	T.N	Avg	MSE	AUROC
**doubleS-GCV**	0.860	0.620	**0.740**	**0.149**	0.834	0.829	0.598	0.714	**0.158**	0.806
AIC	0.685	**0.782**	0.734	1.001	0.771	0.635	0.780	0.708	1.005	0.740
BIC	0.685	**0.782**	0.734	1.009	0.771	0.666	**0.793**	0.730	0.986	0.764
AICC	0.698	0.766	0.732	0.926	0.776	0.667	0.776	0.722	0.779	0.759
HQC	0.932	0.451	0.692	0.581	0.860	**0.959**	0.349	0.654	0.481	0.851
EBIC	0.752	0.717	0.735	0.580	0.796	0.755	0.715	**0.735**	0.541	0.797
EHQC	0.796	0.671	0.734	0.188	0.812	0.797	0.671	0.734	0.177	0.812
Stability Selection	**0.950**	0.417	0.684	0.461	**0.871**	0.941	0.416	0.679	0.446	**0.857**
**Method**	**Type 3**					**Type 4**				
	**T.P**	**T.N**	**Avg**	**MSE**	**AUROC**	**T.P**	**T.N**	**Avg**	**MSE**	**AUROC**
**doubleS-GCV**	0.875	0.610	**0.743**	**0.146**	0.844	0.907	0.602	0.755	**0.145**	0.870
AIC	0.692	**0.786**	0.739	1.078	0.774	0.717	0.782	0.750	0.911	0.789
BIC	0.670	0.778	0.724	1.044	0.764	0.717	0.782	0.750	0.918	0.789
AICC	0.682	0.775	0.729	1.007	0.765	0.729	**0.787**	**0.758**	0.887	0.801
HQC	0.923	0.507	0.715	0.573	0.876	0.961	0.432	0.697	0.294	0.898
EBIC	0.755	0.714	0.735	0.465	0.797	0.755	0.716	0.736	0.540	0.797
EHQC	0.797	0.670	0.734	0.176	0.812	0.798	0.672	0.735	0.182	0.813
Stability Selection	**0.959**	0.411	0.685	0.469	**0.885**	**0.982**	0.416	0.699	0.384	**0.914**

**Table 3:** Performance comparison under the challenging simulation scenario across four types of varying coefficient functions. The reported values are averages over 100 independently generated datasets. Bold values indicate the best performance for each metric within each coefficient-function type.

Method	Type 1					Type 2				
	T.P	T.N	Avg	MSE	AUROC	T.P	T.N	Avg	MSE	AUROC
doubleS-GCV	**0.887**	0.882	0.885	**6.175**	**0.923**	**0.878**	0.882	0.880	**6.256**	**0.917**
AIC	0.879	0.888	0.884	6.276	0.920	0.869	0.888	0.879	6.343	0.914
BIC	0.787	0.942	0.865	7.868	0.880	0.798	0.943	0.870	8.007	0.885
AICC	0.854	0.912	0.883	6.796	0.909	0.842	0.914	0.878	6.995	0.904
HQC	0.839	0.919	0.879	6.982	0.903	0.833	0.920	0.877	7.162	0.900
EBIC	0.736	0.956	0.846	8.782	0.857	0.750	0.957	0.853	8.944	0.864
EHQC	0.787	0.943	0.865	7.872	0.880	0.797	0.943	0.870	8.013	0.885
Stability Selection	0.813	**0.976**	**0.894**	13.045	0.900	0.813	**0.975**	**0.894**	13.493	0.900
**Method**	**Type 3**					**Type 4**				
	**T.P**	**T.N**	**Avg**	**MSE**	**AUROC**	**T.P**	**T.N**	**Avg**	**MSE**	**AUROC**
doubleS-GCV	**0.873**	0.886	0.880	**6.364**	**0.915**	**0.875**	0.882	0.878	**6.163**	**0.916**
AIC	0.865	0.893	0.879	6.464	0.912	0.868	0.888	0.878	6.261	0.914
BIC	0.778	0.944	0.861	8.064	0.875	0.788	0.946	0.867	7.999	0.882
AICC	0.838	0.916	0.877	7.014	0.901	0.840	0.916	0.878	6.935	0.903
HQC	0.826	0.922	0.874	7.183	0.896	0.827	0.922	0.875	7.119	0.898
EBIC	0.723	0.958	0.840	8.976	0.851	0.735	0.959	0.847	8.888	0.858
EHQC	0.777	0.944	0.861	8.068	0.875	0.787	0.946	0.867	8.004	0.882
Stability Selection	0.802	**0.977**	**0.889**	13.387	0.895	0.809	**0.978**	**0.893**	13.509	0.899

**Table 4:** Mean and standard deviation of the mean squared error (MSE) across 20 cell lines. For each cell line and each model selection criterion, a cell line-specific gene regulatory network was constructed by fitting 1000 regression models, one for each target gene. The table reports the mean and standard deviation of the resulting 1000 MSE values. Pairwise Wilcoxon rank-sum tests were performed to evaluate whether the proposed doubleS-GCV method produces significantly smaller MSE values than the competing criteria. *** indicates p<0.0001.

Names of Cell Lines	doubleS-GCV	AIC	BIC	AICC	HQC
CVCL_0079 (cell line 1)	0.608 (0.589) ***	2.349 (3.589)	4.469 (7.473)	4.469 (7.473)	4.469 (7.473)
CVCL_2307 (cell line 2)	1.094 (2.307) ***	2.249 (4.117)	2.372 (4.220)	2.372 (4.220)	2.372 (4.220)
CVCL_1629 (cell line 3)	1.326 (2.535) ***	2.201 (3.805)	2.222 (3.803)	2.222 (3.803)	2.222 (3.803)
CVCL_1073 (cell line 4)	2.129 (4.559) ***	4.524 (7.541)	4.589 (7.559)	4.589 (7.559)	4.589 (7.559)
CVCL_C171 (cell line 5)	1.067 (2.451) ***	2.921 (5.705)	3.033 (5.851)	3.033 (5.851)	3.033 (5.851)
CVCL_1689 (cell line 6)	1.262 (2.725) ***	2.427 (4.401)	2.493 (4.434)	2.493 (4.434)	2.493 (4.434)
CVCL_1207 (cell line 7)	1.977 (4.045) ***	3.163 (5.588)	3.261 (5.750)	3.261 (5.750)	3.261 (5.750)
CVCL_2161 (cell line 8)	1.670 (3.197) ***	4.026 (6.700)	4.169 (6.891)	4.169 (6.891)	4.169 (6.891)
CVCL_2209 (cell line 9)	2.247 (4.030) ***	3.661 (6.261)	3.770 (6.429)	3.770 (6.429)	3.770 (6.429)
CVCL_1475 (cell line 10)	1.121 (3.924) ***	2.887 (5.327)	2.923 (5.352)	2.923 (5.352)	2.923 (5.352)
CVCL_1615 (cell line 11)	1.825 (3.252) ***	2.526 (4.368)	2.555 (4.370)	2.555 (4.370)	2.555 (4.370)
CVCL_1232 (cell line 12)	2.126 (4.088) ***	3.474 (6.272)	3.537 (6.277)	3.537 (6.277)	3.537 (6.277)
CVCL_8792 (cell line 13)	1.842 (3.754) ***	3.551 (6.410)	3.710 (6.607)	3.710 (6.607)	3.710 (6.607)
CVCL_3152 (cell line 14)	1.588 (3.420) ***	2.424 (4.797)	2.505 (4.861)	2.505 (4.861)	2.505 (4.861)
CVCL_1484 (cell line 15)	1.749 (3.397) ***	2.467 (4.803)	2.494 (4.824)	2.494 (4.824)	2.494 (4.824)
CVCL_2961 (cell line 16)	1.656 (3.285) ***	3.414 (5.992)	3.510 (6.077)	3.510 (6.077)	3.510 (6.077)
CVCL_1389 (cell line 17)	1.403 (2.771) ***	3.853 (6.588)	3.904 (6.644)	3.904 (6.644)	3.904 (6.644)
CVCL_1583 (cell line 18)	1.276 (2.140) ***	3.159 (4.965)	3.240 (5.026)	3.240 (5.026)	3.240 (5.026)
CVCL_1348 (cell line 19)	1.331 (2.300) ***	2.402 (4.124)	2.468 (4.165)	2.468 (4.165)	2.468 (4.165)
CVCL_2319 (cell line 20)	0.803 (1.103) ***	2.526 (3.861)	3.202 (6.076)	3.202 (6.076)	3.202 (6.076)

**Table 5:** Candidate biomarkers identified from Cytarabine sensitivity-specific gene network analysis, grouped as shared, resistant-specific, or sensitive-specific genes. Supporting evidence linking these genes to acute myeloid leukemia (AML) and Cytarabine response is summarized.

Category	Gene	AML	Cytarabine
Shared	RPS4Y1	-	-
	LGALS4	[10]	-
Resistant-specific	DDX3Y	[11]	-
	EIF1AY	-	-
	AKR1C1	[12]	-
	HLA-DRA	[13]	-
	HLA-DPA1	[14]	[15]
Sensitive-specific	TYRP1	[16]	-
	GMFG	[17]	[18]
	AEBP1	[19]	-
	CGA	[20]	-
	IGLL1	[21]	-
	MLANA	[22]	-

**Table 6:** Comparison of Cytarabine response-associated network analysis results under different selection criteria and extreme hyperparameter settings. Two cell line-specific networks were estimated under five scenarios: doubleS-GCV, LASSO & small bandwidth, and LASSO & large bandwidth. The following measures were compared: (1) the total number of edges in the estimated network (# edges), (2) the number of edges overlapping with the doubleS-GCV result among the top 1000 edges ranked by absolute weight (# overlapped edges), and (3) the number of genes overlapping with the doubleS-GCV result among genes with node degree ≥5 (# overlapped genes).

Cell Line	Measure	Elastic Net with doubleS-GCV	LASSO & Small Bandwidth	LASSO & Large Bandwidth
CVCL 0079 (cell line 1)	# edges	588,279	7785	864
	# overlapped edges	-	5	3
	# overlapped genes	-	845	133
CVCL 2319 (cell line 20)	# edges	586,747	6568	871
	# overlapped edges	-	46	18
	# overlapped genes	-	987	135

## Data Availability

The code for generating toy data and implementing doubleS-GCV is available on GitHub at https://github.com/JooeeOh/ssGCV.git (accessed on 13 September 2026).

## Appendix A. Local Quadratic Approximation of the *L*_1_-Type Penalty

The local quadratic approximation (LQA) of the $L_{1}$-type penalty is given as follows [9],

$${\left[p(|\beta_{j}|)\right]}^{\prime }=p^{\prime }\left(\right|\beta_{j}\left|\right)\cdot \mathrm{sgn}\left(\beta_{j}\right)=p^{\prime }\left(\right|\beta_{j}\left|\right)\cdot \frac{\beta_{j}}{|\beta_{j}|}\approx \frac{p^{\prime }\left(\right|\beta_{j0}\left|\right)}{|\beta_{j0}|}\beta_{j},\;\beta_{j}\approx \beta_{j0},$$

where $\beta_{j0}$ is an initial value. That is, the penalty $p(|\beta_{j}|)$ is approximated as follows:

(A1) $$\begin{array}{cc} p(|\beta_{j}|)\; & \approx p(|\beta_{j0}|)+{\left[p(|\beta_{j0}|)\right]}^{\prime }\left(\beta_{j}-\beta_{j0}\right)\\ \; & \approx p(|\beta_{j0}|)+\frac{1}{2}\left\{\frac{p^{\prime }\left(|\beta_{j0}|\right)}{\left|\beta_{j0}\right|}\right\}\left(\beta_{j}^{2}-\beta_{j0}^{2}\right). \end{array}$$

## Appendix B. Notation

**Table A1:** Summary of the major symbols used in the model formulation and derivation of doubleS-GCV.

Symbol	Description
*n*	Number of samples or cell lines.
*q*	Number of candidate regulator genes for each target gene.
*ℓ*	Index of the target gene whose incoming regulatory effects are estimated.
α	Index of the target sample for which a sample-specific network is estimated.
$t_{i\ell }$	Expression level of the *ℓ*th target gene in the *i*th sample.
$r_{ij}$	Expression level of the *j*th candidate regulator gene in the *i*th sample.
$m_{i}$	Modulator value of the *i*th sample, such as Cytarabine IC50.
$\beta_{j\ell \alpha }$	Regulatory coefficient from regulator gene *j* to target gene *ℓ* for target sample α.
$\epsilon_{i\ell }$	Random error term, assumed to follow $N(0,\sigma^{2})$.
$K(m_{i}-m_{\alpha }|h_{\ell \alpha })$	Gaussian-kernel weight assigned to sample *i* when estimating the network for target sample α.
R	Design matrix containing the expression levels of the candidate regulator genes.
$K_{\ell \alpha }$	Diagonal matrix of Gaussian-kernel weights for target sample α.
$t_{\ell \alpha }^{*}$	Kernel-weighted response vector, $K_{\ell \alpha }^{1/2}t_{\ell }$.
$R_{\ell \alpha }^{*}$	Kernel-weighted design matrix, $K_{\ell \alpha }^{1/2}R$.
$\Sigma_{\gamma_{\ell \alpha },\delta_{\ell \alpha }}\left({\hat{\beta }}_{\ell \alpha }\right)$	Diagonal penalty matrix obtained using the local quadratic approximation.
$H_{\ell \alpha }$	Hat matrix of the locally weighted regularized model.
$h_{ik}$	(i,k)th element of the hat matrix $H_{\ell \alpha }$.
${\hat{\beta }}_{\ell \alpha }^{\left(-i\right)}$	Coefficient vector estimated after excluding the *i*th observation.
$\hat{df}$	Estimated effective degrees of freedom of the fitted model.
$\mathrm{tr}(H_{\ell \alpha })$	Trace of the hat matrix, representing effective model complexity.
